# Supplementary material for: Are carbon emissions trading and green financial instruments synergistic? -Comprehensive quantitative research based on content analysis
Source: PLoS One. 2024 Mar 7;19(3):e0298601. doi: 10.1371/journal.pone.0298601 (PMC10919601; doi:10.1371/journal.pone.0298601)
Supplement: S1 Data — (ZIP) [file pone.0298601.s001.zip › Data/地级市Z_F工作报告.zip!/║╙▒▒/║Γ╦«╩╨/└┤╫╘╣1⁄2╓┌║┼í╛┬φ┐╦╩2╛▌═°í┐_2004.docx]

各位代表：

现在，我代表枣强县人民政府向大会作工作报告，请予审议，并请县政协各位委员和列席会议的同志提出意见。

2003年工作的回顾

2003年是新一届政府开展工作的第一年，也是全面建设小康社会的起步年。我们在县委的正确领导下，在县人大、县政协的监督支持下，以“三个代表”重要思想为指导，认真贯彻党的十六大精神，团结带领全县人民，开拓进取，扎实工作，战胜非典疫情的冲击，较好地完成了县十三届人大一次会议确定的各项任务目标，全县经济和社会各项事业步入快速发展、良性循环的轨道，实现了全面建设小康社会的良好开局。

经济运行质量提高，发展速度加快。围绕年初确定的“四大战略、六项重点”（强力实施工业强县、民营富县、项目立县、开放兴县四大战略，着力在结构调整、双向开放、项目建设、园区建设、环境建设、县城建设等六个方面实施突破），一以贯之抓落实，千方百计求突破，全县经济呈现出效益提高、速度加快、后劲增强的良好势头。2003年，全县国内生产总值完成40.1亿元，同比增长11.5%；财政收入完成15065万元,同比增长22%。

工业强县迈出坚实步伐，主导产业增势强劲。把玻璃钢、皮毛作为主导产业和立县产业，集中力量，重点推进。玻璃钢业上交税金1800万元，同比增长23%，皮毛业上交税金突破5000万元，比上年翻了一番，有力地拉动了全县经济增长。园区建设进展加快。玻璃钢园区续建和新建项目17个，完成固定资产投资1.1亿元，企业总数达到38家；皮毛工业区新上项目33个，完成固定资产投资1.3亿元，企业总数达到60家。项目建设水平提高。恒润公司的中央空调、恒兴公司的裘皮制品、富顺公司的水貂时装等一批重点项目规模大、起点高，市场前景广阔。骨干企业进一步壮大。新增销售收入超亿元企业6家，全县纳税超百万元的企业达到 15家，亿利莱、兴顺、富邦皮草公司进入全市三十强企业。县乡企业改制扎实推进。已完成改制企业178家，改制面达到75%。通过改组改制,企业活力增强，效益有了新的提高。

农业结构调整扎实推进，农村经济全面发展。红枣业克服严重冻灾的不利影响，一手抓管护，保成活；一手抓育苗，打基础。完成红枣育苗900亩，嫁接枣树157万株，“马脸”牌小枣在国家工商总局成功注册。同时，以确权为突破口，完成植树造林10万亩、1100万株，创历史最高水平，被评为全省植树造林工作先进县。奶牛业以扩充总量、膨胀规模为重点，新建杜烟、王常、唐林、王均等养殖小区4个，全县新增奶牛1000多头，马屯、加会、肖张等3个养殖小区通过了省无公害标准化验收。农业种植结构进一步优化，粮经比例由56：44调整到51：49。全县打更新机井382眼，铺设防渗管道6万米，农业生产条件进一步改善。

发展环境进一步优化，民营经济突飞猛进。制订了鼓励民营经济发展的一系列政策措施,以行风评议为契机，进一步转变作风，改进服务，为民营经济发展排忧解难，调 动了民营企业大干快上的积极性。2003年，全县民营经济完成营业收入62亿元，上交税金8598万元，同比分别增长45%和46 %。一般纳税人由280家一跃发展到410家。民营经济的全面提速，为经济建设和社会发展增添了强大活力。

基础设施日臻完善，县城面貌不断改观。坚持“经营城市”的理念，多方筹措资金，加快县城建设。翻修改造了建设路南关段、市场街中段和北环路，拆迁了人民街西关段，对县城北出口、西出口进行了包装。对枣强商城的商贸用地进行了挂牌出让，对违法占地和违规建筑进行了严厉查处。城网改造工程顺利启动，通讯基站迅速扩展，公路建设不断加快，基础设施得到新的改善。

双向开放取得新成绩，外贸出口快速增长。深入开展了“招商引资年”活动，开放水平不断提高。亚圣、大有等企业与日本、瑞典、意大利等国外客商达成合作意向，对外开放领域进一步扩大。全年新增自营出口权企业16家，总数达到50家，完成外贸出口4023万美元，是去年的6倍。首次由省政府主办的中国？大营第十二届国际皮草交易会取得圆满成功，营皮的知名度得到新的提升，发展空间更加广阔。

财税形势明显好转，金融工作平稳运行。全县经济的快速发展，税收管理的不断规范，促进了财税形势的根本好转。全年完成国税7954万元、地税2251万元，同比分别增长33%和8%。财政收入大幅增加，财政质量明显提高，确保了公教人员工资正常发放、机关正常运转和增资目标的落实。金融部门积极增加小额贷款投放，有力地支持了特色产业和民营经济发展。

抗击非典斗争取得胜利。面对“非典”严峻考验，我们坚决贯彻市委、市政府的决策部署，依靠科学、依靠群众，迅速建立完善了领导、监测、救治“三大体系”，精心组织,严防死守,全县没有出现非典或疑似病例。同时，坚持一手抓非典防治，一手抓经济发展，把非典造成的损失减少到最低限度。

科教文卫等社会事业全面发展。顺利通过了“全国科技进步县”复查验收，共申报科技项目17项，4项被列入国家计划，6项列入省计划。扎实推进了教育教学改革和学校布局调整，顺利通过了省“学前教育工作普及县”验收。在全国率先推行了农村新型合作医疗，农民“因病返贫”问题得到一定缓解。环保和计划生育圆满完成了任务目标。人民武装、社会保障、广播电视、民政、国土、文化、体育、统计、审计、物价、档案等社会各项事业取得新进展。

精神文明建设和民主法制建设进一步加强。深入开展了“学《纲要》、讲文明、树形象”活动、“文化示范村”创建活动和“改陋习、树新风、建设美好家园”活动，有力地提高了广大群众的公德意识、环境意识和社会责任意识。积极推进民主决策，自觉接受人大的法律监督、工作监督和人民政协的民主监督，人大代表建议和政协委员提案办理质量不断提高，按时办结率达到100%。全面加强社会治安综合治理，不断强化信访工作，认真开展矛盾纠纷排查，有效地化解了各种不安定因素。扎实开展严打整治斗争，有力地打击了各种违法犯罪活动。全县政治安定、社会稳定的大好局面进一步巩固。

各位代表，经过全县上下的共同努力，全县经济和社会各项事业都呈现出可喜的发展局面，形势令人鼓舞，成绩来之不易。这是县委正确领导，人大、政协积极监督、鼎力支持的结果；是全县各条战线、各界人士、广大干部群众团结协作、艰苦奋斗的结果。在此，我代表县政府向在座的同志们，并通过你们向全县各行各业的干部职工、农民群众、知识分子和所有关心、支持、帮助我县发展的朋友们、同志们，表示崇高的敬意和衷心的感谢！

在肯定成绩的同时，我们也清醒地看到，我县经济和社会发展中还存在一些亟待解决的矛盾和问题。与先进地区比，经济总量小，综合实力还不够强；农业主导产业规模小，产业化程度低，农民收入增长缓慢；立县立乡的大项目少，骨干企业少，工业强县的任务还十分艰巨；对外开放水平不高，招商引资工作有待加强；城镇化水平较低，基础设施建设相对滞后；制约经济发展的思想障碍和体制障碍依然存在，发展环境还不尽如人意，干部队伍的工作作风、办事效率与人民群众的要求还有一定差距。对此，我们将高度重视，采取有针对性的措施，认真加以解决。同时，也恳请各位代表、政协委员多提批评和建议。

2004年工作的指导思想和主要任务

2004年是保持枣强发展良好势头，加快全面建设小康社会进程的关键一年。从大的外部环境看，世界经济复苏加快，国内经济升温加热，利用“两种资源、两个市场”机遇增多，空间加大；从我们自身看，枣强经济迎来了新的发展时期，无论速度、效益、活力、后劲都具备了进一步加快发展的基础和条件。同时，也必须看到，区域经济竞争更加激烈，各地发展的热潮一浪高过一浪，我们面临着不进则退、慢进亦退的挑战和压力。必须增强危机感、紧迫感，变有利形势为发展优势，变竞争压力为发展动力，努力实现经济建设和各项事业的新突破。

今年政府工作总的指导思想是：以邓小平理论和“三个代表”重要思想为指导，深入贯彻落实党的十六大和十六届三中全会精神，以全面建设小康社会总揽全局，坚持既定的工作思路和工作重点不动摇，按照“思想再解放，发展再加快，机制再求活，工作再做实”的总体要求，突出“四大战略”，狠抓“五项重点”，把各项工作往深里做，往实里做，推动国民经济持续快速协调发展和社会全面进步。

“四大战略”就是继续深入实施工业强县、民营富县、项目立县和开放兴县战略。实践证明，工业是强县之本，民营是富县之源，项目是立县之基，开放是兴县之举。加快枣强发展，必须牢牢把握四大战略不动摇。

“五项重点”：一是特色经济。从一定意义上讲，区域经济的竞争就是特色经济的竞争。在加快玻璃钢、皮毛、红枣、奶牛四龙经济发展的同时，积极培育新的特色产业，构筑以四大龙型经济为主，其他特色产业为辅的特色经济发展格局。二是园区经济。园区建设是以工强县的主要依托，是现代工业发展的必然选择，也是招商引资不可缺少的平台。必须举全县之力促进工业园区上规模、上水平，使之成为枣强经济的重要支撑和枣强形象的新亮点。三是民营经济。近几年枣强发展的最大受益在于民营经济，必须进一步完善政策，让民营经济更加放心、放胆、放手发展，迸发出更大的活力，加快富民强县进程。四是开放经济。开放是加快发展最直接、最有效、最快捷的途径，开放的力度有多大，发展的速度就会有多快，必须把开放贯穿到经济工作的各个方面，加大推动力度，开创招商引资新局面。五是城镇经济。加强城镇建设、发展城镇经济是统筹城乡协调发展、统筹三次产业协调发展的战略举措，也是社会文明进步的重要标志。必须按照城镇经济与城镇建设协调并进、良性互动的原则，大力推进城镇化进程。

今年的主要预期目标是：国内生产总值增长11.5% 以上，财政收入增长11%以上，全社会固定资产投资增长20 %。

实现上述目标，必须重点抓好以下七个方面的工作：

一、以皮毛、玻璃钢两大产业为龙头，以民营经济为主体，以园区建设为重点，全力推进工业经济再上新台阶

工业是国民经济的主导，是财政收入和城乡居民收入的主要来源，没有工业化，就没有农村的城镇化和农业的现代化。工业不强，加快发展就是一句空话。必须把工业做为经济工作的“第一要务”，在全县上下进一步唱响以工强县主旋律，营造大上工业、上大工业的浓厚氛围。

突出主导产业,打造发展亮点。皮毛产业抓好营皮北扩，膨胀群体规模；启动皮毛市场建设，培育新的增长点；采用先进工艺，改进硝染、鞣制技术；壮大骨干企业，增强龙头带动力；实施名牌战略，抢占国内、国际市场制高点。力争实现“54321”的发展目标，即年内建成销售收入超亿元的企业5家，行业销售收入突破40亿元，固定资产投入达到3个亿，完善建设两个市场（大营集贸市场和皮毛交易市场），出口创汇力争达到1亿美元。通过2到3年的努力，把皮毛业打造成为全市第一产业。 玻璃钢产业增上中央空调主机、陶瓷刮刀等新产品，调整产品结构，拓展应用领域，提高科技水平，增强市场竞争力。力争把玻璃钢生产力促进中心建成国家级生产力促进中心。全年完成销售收入28亿元，建成销售收入超亿元企业6家，努力在群体规模、企业素质、市场份额、实现利税上取得历史性突破。

加快园区建设，做大发展载体。按照现代工业园区的要求，进一步搞好规划设计，完善功能分区。加强绿化、美化、硬化等基础设施建设，拉大园区框架。以优美的环境、优质的服务吸引大企业、大财团入园投资建厂。玻璃钢园区完成东环路、富强路的向北延伸及北环路、平原街的硬化、绿化、美化。力争新进企业20家，完成固定资产投资2.8亿元，建成投资超千万元的企业8家；大营皮毛工业区重点向东、向北延伸，加快中小企业入区步伐。全年确保新进企业30家，完成固定资产投资2.6亿元，建成投资2000万元以上的企业2家，500万元以上企业8家。通过2？3年的努力，把两个工业区建成国内一流、国际知名的生产、研发、销售基地。

壮大民营经济，做强发展主体。一是完善政策，激励发展。进一步完善民营经济发展的各项政策措施，真正做到想民营企业所想，急民营企业所急，帮民营企业所需，切实解决障碍民营经济发展的各种问题。二是选准突破口，推动发展。把四大产业作为民营经济发展切入点的同时，对托辊、印刷、液化气配件、电线电缆、水暖铸造等特色产业，加强研究，认真谋划，寻求突破。三是加强引导，促进发展。引导民营企业搞好科技创新、管理创新和产品创新，加速规模膨胀，增强市场竞争力。通过努力，力争民营经济发展速度保持在30%以上。

搞好县乡企业改制。继续按照“一退两转三买断”的原则，严格程序，规范运作，积极稳妥，全面推进，到年底前全部完成改制任务。引导改制企业进一步规范内部管理，增加投入，加快发展。

二、以农民增收为核心，以高效农业为重点，推动农业结构调整迈出新步伐

深入贯彻中央农村工作会议精神，更加突出地关注农村，支持农业，关心农民，千方百计拓宽农民增收空间。

继续大力发展红枣、奶牛两大特色产业，加快农业产业化进程。红枣业一方面加强现有枣树的规范管理，确保成活率，力促早结果、早见效；另一方面，加大嫁接和育苗力度，全年新植枣树100万株，嫁接500万株，育苗800亩。同时，以确权为突破口，突出抓好植树造林，着力实施“两河十渠”补空造林、乡村主干路绿化、村庄绿化和育苗四大工程。全年造林达到5万亩，植树300万株，育苗800亩。奶牛业按照“现有小区抓完善，新建小区抓进度”的思路，扩张养殖总量，年内新建养殖小区4个，全县奶牛存栏确保5000头，力争6000头。围绕两大产业发展，突出做好三项工作：一是进一步搞好宣传发动。通过典型引路、现身说法等多种形式，使广大农民群众真正认识到两大产业的效益和优势，增强发展的自觉性、主动性，使之真正成为千家万户的产业。二是加强防疫灭病体系建设，积极探索奶牛保险的有效途径，解除农民的后顾之忧，调动发展两大产业的积极性。三是充分发挥林业、畜牧等部门的职能作用，有针对性地搞好技术指导和销售服务，切实帮助群众解决各种实际问题，促进两大产业的快速发展。

在重点抓好两大产业的同时，因地制宜，发展其它效益好的养殖业和棉花、果品等高效经济作物，积极推广优质小麦种植，促进农业结构的全面调整。

提高农业的市场竞争力。抓好农业科技示范区建设，加快农业先进实用技术推广应用，改良品种，提高产量。组织好果品、畜禽产品的标准化生产。以信息、销售、科技为重点，加强农村社会化服务体系建设。大力发展各种类型的农民合作组织，引导农民调结构、拓市场、促增收。

加强农田水利基本建设。坚持开源与节流并重，全年新打深浅机井300眼，铺设防渗管道10万米，新建咸淡水混浇井组200个。继续抓好引黄配套工程，增强引蓄水能力。进一步加大农业综合开发力度，争取更多的项目，提高开发水平。

认真落实上级扶持“三农”（农业、农村、农民）的各项政策，切实减轻农民负担。积极发展劳务输出，拓宽输出渠道，提高输出水平。

三、大力开展招商项目年活动，开创招商引资和项目建设新局面

浓厚开放氛围。按照“氛围再浓厚、思路再拓宽、责任再强化”的要求，完善各项政策措施，进一步把各级干部和企业推向招商引资主战场，构筑起领导带头、部门联动、企业主动、全民参与的开放工作新格局。

拓展招商领域。抓住南资北移、南企北移的机遇，组织开展外出考察招商活动，加快产业对接，拓宽投资渠道。在搞好皮毛企业赴港、赴京参展招商的同时，力争组团赴欧洲参观参展，提高营皮知名度。办好中国？大营第十三届国际皮草交易会，力争取得更加丰硕的成果。

扩大外贸出口。适应国际市场形势变化，加强外贸人才建设，拓宽出口领域，打造出口品牌，在巩固扩大皮毛、化工等优势产品出口的同时，大力开发新的出口产品。力争全年新增自营出口企业20家，实现外贸出口1亿美元。

抓好重点项目。一方面，抓好谋划和前期准备工作。着力抓好富达集团新上中央空调生产流水线、亚圣公司新上陶瓷刮刀、利升皮草公司、亿利莱皮草公司新上高档裘皮服装等14个投资超千万的重点项目。实行县领导、主管部门、金融单位、项目法人“四位一体”责任制，建立专门班子，全力跑办，落实建设条件。另一方面，抓好在建项目。对恒信公司新上轻钢生产流水线、三阳盛业公司新上模压制品、福顺皮草公司新上裘皮制品等7个投资超千万元的在建项目，倒排工期，列表上墙，挂图作战，确保工程质量和建设进度。

加强环境建设。当前，区域经济的竞争集中表现为发展环境的竞争。环境问题已成为影响经济发展的关键因素，发展环境的优劣直接决定着开放的水平、发展的速度和经济的活力。必须牢固树立环境是“第一竞争力”的观念，营造创业光荣、成业有功，亲商重商、扶商护商的良好环境。在完善硬环境的同时，重点优化软环境，积极推行“项目代理帮办”、“服务承诺”、“限时办结”等制度。大力开展企业评部门活动，对破坏环境的行为，严肃查处，把枣强打造成政策最优、环境最好、服务最佳、效率最高的投资宝地。

四、统筹城乡发展，打造城镇建设新亮点

搞好规划建设。按照“北移西扩”的县城布局，以北环路为景观轴线，以玻璃钢园区和文化活动中心为主体，规划建设集行政、居住、休闲、文体、商贸于一体的县城新区。搞好绿宝公司南水塘、县城东南环保湖、环城水系、卫千渠、索泸河沿岸绿化带、千亩森林公园的规划控制，逐步实现绿化美化，提高县城品位。

完善县城功能。规划建设县城集贸市场和建材市场；抓好植物公园、住宅小区的启动建设；以武馆线建设为契机，拓宽改造建设北路，高标准建好县城迎宾路。加快县城东、西出口改造，提升县城形象。

强化县城管理。严格落实县城规划，坚决杜绝乱占乱建。引入市场机制，搞好县城卫生管理。深入开展环境卫生综合整治，不断强化全民城市意识。

提高城镇化水平。以县城、大营镇为重点，以玻璃钢、皮毛两大产业为支撑，加快基础设施建设，加快产业聚集和人口聚集，壮大城镇经济，膨胀城镇规模，努力把县城和大营镇建成功能健全、环境整洁，具有较强辐射带动能力的农村区域性经济文化中心。

五、坚持稳中求活，实现财税金融工作的新提高

在全县财税状况明显好转的新形势下，对优势产业、骨干企业、民营经济进一步给予重点扶持，壮大财源税基。坚持依法治税，确保应收尽收。认真落实财政综合预算、集中支付、政府采购和收支两条线制度，强化预算外资金管理。严格支出顺序，确保公教人员工资发放，确保机关正常运转。积极探索联户贷款、信用贷款等有效途径，大力推广小额信贷，扩大信贷投放，实现金融和地方经济的相互促进。

六、坚持经济与社会协调发展，确保各项事业取得新进展

大力发展科教文卫事业。完善技术创新体系，搞好科技成果转化，加快培育科技型企业。继续深化教育体制改革，提高教学质量。抓好科技、文化、卫生“三下乡”，增强群众科技文化素质。进一步规范新型农村合作医疗，建立健全疾病预防控制体系，促进卫生事业发展。

做好社会保障工作。健全完善社会保障体系，扩大养老、失业保险覆盖面，完善医疗保险制度，巩固“两个确保”，搞好“三条保障线”的衔接，积极推行农村最低生活保障制度，切实保障弱势群体的基本生活。

推进民主法制建设。自觉接受人大的法律监督、工作监督和政协的民主监督，推进决策民主化、科学化。加强基层民主政治建设，大力推行“四公开”，搞好“四五”普法，提高全民法制观念。

全力维护社会稳定。深入开展严打斗争，严厉打击各种犯罪活动。坚决打击“法轮功”等邪教组织，做好教育转化工作。切实搞好社会治安综合治理，抓好信访和矛盾纠纷排查，及时消除不稳定因素。整顿和规范市场经济秩序，净化市场环境。认真抓好安全生产，防止各类事故发生，确保人民生命财产安全。

此外，进一步做好计划生育、人民武装、广播电视、民政、人事、档案、审计、统计、体育等各项工作，促进经济与社会的协调发展。

七、加强自身建设，树立政府新形象

面对新形势、新任务，必须按照“为民、务实、清廉”的要求，切实加强政府自身建设，加快推进政府职能转变和管理创新，建立服务型政府。

解放思想，更新观念。重点强化四个意识：一是发展意识。发展是枣强人民的最高利益，一切为了发展，一切围绕发展，一切服从于发展，坚决清除不利于发展大局的各种障碍；二是机遇意识。敏锐地发现机遇，果断地抓住机遇，创造性地用好机遇，因势而动，乘势而上；三是竞争意识。市场经济就是竞争，必须研究竞争，勇于竞争，善于竞争，努力做到先人一步、高人一筹，夺取主动；四是创新意识。紧跟时代节拍，做到与时俱进，大胆实践，大胆创造，不断开创各项工作新局面。

转变职能，改进作风。按照市场经济的要求，切实把政府职能转到主要为市场主体服务和创造良好发展环境上来。大力弘扬求真务实作风，在调查研究上下功夫，在解决实际问题上下功夫，在“严细深实快”上下功夫。加快行政审批制度改革，提高办事效率，增强工作透明度。加强纪律建设，确保政令畅通。加强公务员学习培训，提高公务员素质。

廉洁自律，从严治政。时刻牢记“两个务必”，始终保持谦虚、谨慎、不骄、不躁的作风，始终保持艰苦奋斗的作风，认真落实党风廉政建设责任制，做到组织领导到位、责任落实到位。对政府采购和政府投资的公益性项目，全面实行公开招投标制度，招投标情况和项目竣工后的审计情况及时向社会公布，接受群众监督，努力从源头上预防和治理腐败，树立良好的政府形象。

勤政为民，多办实事。把亲民、爱民、为民作为工作的出发点和归宿点，一切为了群众，一切依靠群众，察民意，虑民计，为民生，谋民利，从群众最关心的事情抓起，切实把人民群众的利益实现好、维护好、发展好。今年，努力做好以下几件实事：1、启动县城新区规划建设；2、完成富强路、人民街包装工程；3、扩展改造文化休闲广场、火车站广场，完善市政设施建设，逐步解决“如厕难”问题；4、启动大营皮毛交易市场建设；5、完成宽带网乡乡通工程；6、完成枣强县疾病控制中心建设；7、力争完成县城电网建设和改造工程；8、开工建设武馆线、邢德路枣强段加宽改建工程，做好肃临线枣强段加宽改建工程的前期准备工作，力争开工建设；9、翻修改造人民街西关段和县城南北铁路立交桥主路面。

各位代表，新的形势催人奋进，新的任务光荣艰巨。让我们在市委、市政府和县委的正确领导下，高举邓小平理论伟大旗帜，深入贯彻“三个代表”重要思想，团结一致，锐意创新，奋力拼搏，为实现枣强更快更好的发展做出我们新的贡献！
